# Oxidative Stress and Antioxidant Therapy in Pulmonary Hypertension

**DOI:** 10.3390/antiox12051006

**Published:** 2023-04-26

**Authors:** Paula Poyatos, Miquel Gratacós, Kay Samuel, Ramon Orriols, Olga Tura-Ceide

**Affiliations:** 1 Department of Pulmonary Medicine, Dr. Josep Trueta University Hospital de Girona, Santa Caterina Hospital de Salt and the Girona Biomedical Research Institute (IDIBGI), 17190 Girona, Spain; ppoyatos@idibgi.org (P.P.); mgratacos@idibgi.org (M.G.); 2 Department of Medical Sciences, Faculty of Medicine, University of Girona, 17003 Girona, Spain; 3 Scottish National Blood Transfusion Service, NHS National Services Scotland, Edinburgh EH14 4BE, UK; 4 Biomedical Research Networking Centre on Respiratory Diseases (CIBERES), 28029 Madrid, Spain

**Keywords:** pulmonary hypertension, oxidative stress, antioxidant therapy, endothelial dysfunction

## Abstract

Pulmonary hypertension (PH) is a progressive disease characterized by elevated artery pressures and pulmonary vascular resistance. Underlying mechanisms comprise endothelial dysfunction, pulmonary artery remodeling and vasoconstriction. Several studies have shown evidence of the critical role of oxidative stress in PH pathophysiology. Alteration of redox homeostasis produces excessive generation of reactive oxygen species, inducing oxidative stress and the subsequent alteration of biological molecules. Exacerbations in oxidative stress production can lead to alterations in nitric oxide signaling pathways, contributing to the proliferation of pulmonary arterial endothelial cells and smooth muscle cells, inducing PH development. Recently, antioxidant therapy has been suggested as a novel therapeutic strategy for PH pathology. However, the favorable outcomes observed in preclinical studies have not been consistently reproduced in clinical practice. Therefore, targeting oxidative stress as a therapeutic intervention for PH is an area that is still being explored. This review summarizes the contribution of oxidative stress to the pathogenesis of the different types of PH and suggests antioxidant therapy as a promising strategy for PH treatment.

## 1. Pulmonary Hypertension

Pulmonary hypertension (PH) is a progressive disease characterized by increased pulmonary vascular resistance and pulmonary artery pressures [1]. The hemodynamic definition of PH has recently been updated and is currently defined as an increase in mean pulmonary arterial pressure (mPAP) >20 mmHg at rest [2]. This chronic pressure overload due to PH, leads to the development of right ventricular hypertrophy (RVH), heart failure and, ultimately, death [1,3]. Structural remodeling of the vasculature, resulting in reduced vessel lumen, is related to increased pulmonary vascular resistance and increased pulmonary pressure [1].

Clinical classification of PH categories clinical conditions associated with PH into five groups according to similarities in clinical presentation, pathological findings, hemodynamic characteristics, and therapeutic management (Table 1) [4]. Group I is a rare condition known as pulmonary arterial hypertension (PAH), with a prevalence of 48–55 cases per million adults [4]. PAH can be idiopathic (iPAH), heritable (hPAH), drug- and toxin-induced, associated with several conditions or diseases, with features of venous/capillary involvement or persistent PH of the newborn, with iPAH being the most common subtype (50–60% of all cases) [4]. hPAH includes patients with mutations in BMPR2 (bone morphogenetic protein receptor type 2), a member of the transforming growth factor (TGF-β) superfamily, seen in 70–80% of hPAH [5]. Group II PH or left heart disease (PH-LHD) is the main cause of PH, accounting for 75% of all cases of PH [6]. It is caused by increased left atrial pressure, normally occurring as a consequence of an underlying cardiac disorder [7]. In group III, PH is related to lung disease or hypoxemia. Chronic obstructive pulmonary disease (COPD) is the most common lung disease associated with PH, accounting for about 80% of cases [8]. Group IV, chronic thromboembolic PH (CTEPH), is a progressive disease caused by the obstruction of major pulmonary arteries as a consequence of flow-limiting organized thrombi [9]. Finally, group V encompasses a complex group of disorders associated with PH. The cause can be related to multifactorial mechanisms and can be secondary to increased pre- and post-capillary pressure or direct effects on pulmonary vasculature [4].

## 2. Oxidative Stress

Aerobic metabolism involves the production of reactive oxygen species (ROS), even under basal conditions, where it plays an essential role in some physiologic signaling pathways, such as inflammation. Therefore, there is a continuous requirement for the inactivation of these reactive oxygen species [10]. The term ROS describes a variety of small molecules characterized by high reactivity and biological activity. It mostly includes superoxide anion (O2^•−^), hydroxyl radical (OH^•^), hydrogen peroxide (H_2_O_2_), singlet oxygen (^1^O_2_), peroxynitrite (ONOO^−^), or hypochlorous acid (HOCl) [11]. ROS can be generated from various sources. In human cells, there are several H_2_O_2_ and O_2_^−^ generating enzymes, the NADPH oxidases (NOXs) the major endogenous enzymatic source of H_2_O_2_ and O_2_^−^ together with the mitochondrial electron transport chain (ETC) [12]. Mitochondrial ROS (mtROS) is generated as a consequence of electron transfer during ATP production. Electrons that leak out of the ETC at complex I and III, can react with oxygen producing O_2_^−^ [13]. Apart from NOXs and the mitochondrial ETC, H_2_O_2_ can be generated by oxidase enzymes found in other subcellular locations, mainly in the endoplasmic reticulum (ER) and peroxisomes [12]. Several superoxide dismutases (SOD1–SOD3) can also produce H_2_O_2_ from O_2_^−^ [12,13]. In addition to intracellular sources, ROS can be also generated by cumulative environmental exposure, such as molecular factors (drugs, pollution and nutrients), physical (UV, X-ray and other ionizing radiation), and psychological stressors (lifestyle) [12].

The excessive production of ROS associated with mitochondrial, enzymatic, or exogenous ROS sources can result in an imbalance between ROS production and the cells’ defense systems, inducing oxidative stress, resulting in subsequent alteration of biological molecules, including DNA, lipids, proteins, and carbohydrates [10,13]. Consequently, oxidative stress could be involved in processes such as mutagenesis, carcinogenesis, membrane damage, lipid peroxidation, protein oxidation and fragmentation, carbohydrate damage, as well as in the pathogenesis of several diseases [10,13].

The following sections will describe in detail the published evidence of oxidative stress in the different subtypes of PH.

## 3. Oxidative Stress in the Different Subtypes of PH

Several studies have shown evidence of oxidative stress in the lungs and pulmonary vasculature of animals and humans with PH (Table 2).

### 3.1. Oxidative Stress in PAH (Group I PH)

**Figure 1:**
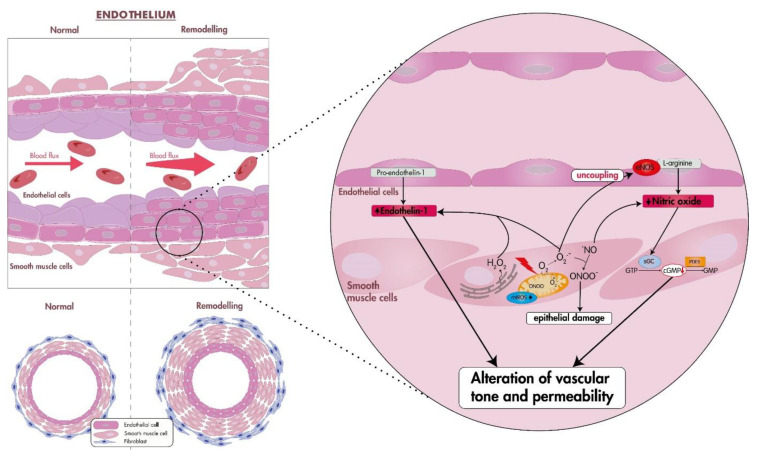
Effects of oxidative stress in the vascular endothelium. Oxidative stress plays a major role in the development of endothelial dysfunction and pulmonary hypertension, producing an imbalance between the synthesis and release of endothelium-derived constrictor factors such as endothelin-1 and a decrease in vasodilators factors such as nitric oxide, contributing to pulmonary artery remodeling, vasoconstriction, and leading to the alteration of vascular tone and vascular permeability. Reduction in endogenous NO levels is achieved when reactive oxygen species, specially O^2−^, react with NO producing the intermediate peroxynitrite, causing epithelial damage. Abbreviations: H_2_O_2_, hydrogen peroxide; O^2−^, superoxide anion; ONOO^−^, peroxynitrite; NO, nitric oxide; mtROS, mitochondrial ROS; eNOS, endothelial nitric oxide synthase; sGC, soluble guanylate cyclase; cGMP, cyclic GMP; PDE, phosphodiesterase.

Vasoconstriction promoted by oxidative stress is probably one of the most critical factors in the early stages of PAH [14]. Oxidative stress plays a key role in impairing endothelial cell function, producing an increase in the synthesis and release of endothelium-derived constrictor factors such as endothelin-1 (ET-1) and a decrease in relaxing factors such as NO, contributing to the alteration of vascular tone and vascular permeability [15,16]. Reduction in endogenous NO levels, an important signaling molecule involved in the modulation of vascular tone, blood pressure, and the regulation of smooth muscle proliferation and migration, may contribute to the development of PAH [14,17] (Figure 1). This reduction in NO bioavailability is achieved when ROS, principally O_2_^−^ that reacts readily with NO, forms the intermediate peroxynitrite (ONOO^−^), which reacts with available tyrosine residues of proteins producing 3-nitrotyrosine, causing lung epithelial damage [18,19]. Therefore, most animal models of PH aim to reproduce the two principal pathological characteristics in the pulmonary vasculature, common to most PH groups, which are excessive vasoconstriction and pulmonary vascular remodeling (PVR) [20].

In animal studies, Guo et al. [21] found that in monocrotaline (MCT)-treated rats, which develop severe PAH, the observed increased oxidative stress caused decreased pyruvate kinase isoenzyme type M2 (PKM2) activity, resulting in increased proliferation of pulmonary artery smooth muscle cells (PASMCs). Moreover, to confirm that PKM2 was triggered by ROS, they treated MCT-PAH rats with the antioxidant N-acetylcysteine (NAC), showing an attenuation of PKM2 activity, thus demonstrating the role of ROS in cell signaling for the pathogenesis of PAH.

In human studies, Sun et al. [22] showed that monoamine oxidase (MAO), an important ROS source implicated in different vascular diseases, specifically MAO-A expression, was increased in the medial and intimal layers of patients with PAH. They also determined that this increase was involved in the progression of PAH and that MAO-A inhibitors could reverse PVR.

Similarly, Cracowski et al. [23] established that urinary levels of isoprostaglandin F_2α_ type III (iPF_2α_-III), a stable and specific product of lipid peroxidation, were 2.3 times higher in patients with PAH and other types of PH than in healthy controls, showing that oxidative stress is increased in patients with PH.

Other studies have shown increased oxidative stress levels in persistent PH of the newborn (PPHN). Brennan et al. [24] demonstrated increased superoxide formation, without a simultaneous increase in cellular antioxidant capacity, in PPHN lungs compared with controls. In addition, elevated levels of H_2_O_2_ in PPHN pulmonary arteries, associated with decreases in cGMP signaling, have been shown to contribute to development of the pathology [25].

### 3.2. Pulmonary Hypertension Caused by Left Heart Disease (Group II PH)

PH-LHD is the most common form of PH, accounting for 65–80% of all cases [26,27]. PH-LHD develops mainly due to the sustained elevation of left-sided filling pressure because of left-ventricular (LV) dysfunction, producing an increase in pulmonary arterial pressure and subsequently pulmonary vascular remodeling [28]. This pulmonary venous congestion may promote additional pathophysiological changes, such as pulmonary vasoconstriction, decreased NO availability, increased expression of ET-1 and desensitization to natriuretic peptide-induced vasodilation [26,27].

Several studies have demonstrated increased oxidative stress in patients with PH, but there is little evidence of oxidative stress in PH group II.

Ravi et al. [28] found high levels of peroxynitrite and superoxide in left-heart failure induced rats, and showed that this mediated the downregulation of PTEN expression, a phosphatase-and-tensin homolog on chromosome 10 and a modulator of the phosphoinositide 3-kinase activity related to vascular remodeling. Decreased PTEN expression resulted in smooth muscle cell (SMC) proliferation and subsequent vascular remodeling, demonstrating the association between oxidative stress and the pathogenesis of PH-LDH. Using a synthetic analogue of curcumin, HO-3867, a molecule with antioxidant and antiproliferative properties, they showed significant attenuation of oxidative stress, resulting in upregulation of PTEN expression and inhibition of vascular remodeling.

Furthermore, Sunamura et al. [29] also demonstrated that ROS was involved in the pathogenesis of PH-LDH. They found that mice deficient in ROCK1 (cROCK1^−/−^), a rho-kinase member of the serine/threonine protein kinase family, showed pressure-overload-induced cardiac dysfunction and postcapillary PH. Additionally, they showed upregulation of ROS levels by cyclophilin A (CyPA) and basigin (Bsg), two common molecules that augment heart failure (HF) and PH. However, downregulation of ROS and CyPA and Bsg proteins has also been reported in cROCK2^−/−^ mice with attenuated cardiac dysfunction and postcapillary PH. Moreover, ROCK1 deficiency destroyed the balance between mitochondrial fission and fusion, resulting in impaired mitochondrial homeostasis and the generation of ROS. Interestingly, upregulation of ROCK2 in cROCK1^−/−^ mice produced an increase in ROS levels after pressure overload, showing that ROCK2 specifically increases ROS production mediated by the downstream effectors CyPA and Bsg. Additionally, celastrol, a compound with antioxidant and anti-inflammatory effects, reduces CyPA and Bsg expression, reducing ROS production and improving pressure-overload-induced cardiac dysfunction and postcapillary PH, showing the role of oxidative stress in the development of PH-LHD.

### 3.3. Pulmonary Hypertension Caused by Lung Diseases and/or Hypoxia (Group III PH)

PH associated with hypoxia and lung disease is the second most common type of PH. The most common lung diseases causing PH are COPD and interstitial lung disease, but it can also be associated with other pathologies such as cystic fibrosis and high altitude exposure [30]. The origin of PH in hypoxic lung disease is multifactorial. Chronic lung diseases produce periods of continuous or intermittent hypoxia, increasing the release of vasoconstrictors causing pulmonary artery vasoconstriction (HPV), leading to vascular remodeling, which increases vascular resistance and pulmonary artery pressure [30,31]. Moreover, under hypoxic conditions, the expression of oxidative stress biomarkers increases, causing cellular damage [31].

Liu et al. [32] demonstrated that increased generation of O_2_^−^ or ROS derived from this anion acting on PASMCs was required for HPV. Treatment of distal porcine pulmonary artery PASMCs with SOD or SOD with catalase (CAT), inhibited constriction induced by hypoxia, suggesting that ROS play an essential role in HPV during hypoxia. Weissmann et al. [33] provided evidence that superoxide formation, perhaps derived from a NAD(P)H oxidase, and the subsequent generation of H_2_O_2_ is the underlying mechanism of acute HPV, having a role in the signaling cascade linking hypoxia sensing and vasoconstrictor phenomena and suggesting that hypoxia produces an increase rather than a decrease in ROS levels.

Many studies have shown that hypoxic exposure might cause oxidative stress in lung tissue. Hoshikawa et al. [34] found that lung tissue levels of PCOOH, a primary peroxidation product of phosphatidylcholine, increased after hypoxic exposure, and that administration of the antioxidant NAC reduced hypoxia-induced cardiopulmonary alterations and inhibited the increase in PCOOH levels. Inhibition of xanthine oxidase (XO)-hypoxanthine, an important pathway in generating oxidative stress in vivo, decreased PCOOH levels in hypoxia exposed rats, which showed attenuation of pulmonary hypertension, right ventricular hypertrophy, and pulmonary vascular media thickening.

In a hypoxia-induced PH model You et al. [35] observed increased NOX4 and VPO1 expression, as well as HOCl production. VPO1, a member of the peroxidase family, uses chloride and NOX-derived H_2_O_2_ to produce HOCl, a more potent oxidizer that accelerates the increase of oxidative stress in the vasculature. They also observed enhanced proliferation, apoptosis resistance, and migration of PASMCs, demonstrating that NOX4/VPO1 pathway-mediated oxidative stress promotes vascular remodeling.

Furthermore, Pu et al. [36] reported that chronic high-altitude exposure produced pulmonary hypertension, increasing the generation of both malondialdehyde (MDA) and ROS, and decreasing glutathione peroxidase and SOD activities, which was accompanied by pulmonary vessel remodeling.

### 3.4. Oxidative Stress in CTEPH (Group IV PH)

CTEPH is produced by unresolved blood clots associated with fibrosis that obstruct pulmonary arteries [37]. However, the mechanisms leading to the lack of pulmonary thrombo-emboli resolution remain unclear [38]. It has been suggested that vascular dysfunction, such as endothelial dysfunction, caused by a decrease in NO availability due to the overproduction of ROS, may contribute to the progression of the pathology [39,40].

CTEPH-derived endothelial cells (CTEPH-EC) presented a significant increase in oxidative stress levels, specifically mtROS production, and reduced expression of SOD-2 compared to healthy human pulmonary artery ECs used as control cells [41]. Nukala et al. also showed that CTEPH-EC exhibited an increase in intracellular ROS, advanced oxidation protein products and total protein carbonyl content (PCO), and a downregulation of GPX4 and GPX1 proteins, demonstrating dysregulation of the oxidative stress response and highlighting the involvement of oxidative stress in CTEPH [42].

Brandt et al. [38] found that expression of NADPH oxidase and superoxide formation increased in mice with induced PE, resulting in endothelial dysfunction in pulmonary arteries. Furthermore, Stam et al. [43] using a swine-CTEPH model, in addition to other changes, observed increased expression of genes associated with oxidative stress (ROCK2, NOX-1, and NOX-4) in the right ventricle (RV) which contributed to RV hypertrophy and dysfunction.

In another study, Smukowska-Gorynia et al. [37] evaluated antioxidant status in patients with deteriorating or stable CTEPH, analyzing serum oxidative stress biomarkers including: MDA, a lipid peroxidation indicator; total antioxidant capacity (TAC), for the evaluation of antioxidant status; and CAT and SOD activities, two of the main cellular antioxidant systems. MDA was higher in the deteriorating group compared with stable patients, while TAC and CAT were lower, with no significant difference in SOD, suggesting that MDA concentration and TAC and CAT activities correlated with adverse clinical outcomes, showing an imbalance between the generation of ROS and the biological detoxification system.

Zhang et al. [44] measured oxidative-antioxidant biomarker levels, and asymmetric dimethylarginine (ADMA), an endogenous NO synthase inhibitor implicated in some cardiovascular diseases, to assess the association of these biomarkers with the development and prognosis of CTEPH. Comparing healthy controls and CTEPH patients, they found a significant increase in MDA and ADMA levels in the CTEPH group, as well as an important decrease in the biological antioxidant potential and SOD levels, suggesting that ROS contributes to the pathogenesis of CTEPH.

### 3.5. Pulmonary Hypertension with Unclear and/or Multifactorial Mechanisms (Group V)

Group 5 PH includes several diseases in which the mechanisms leading to the development of PH are unclear [45]. PH can be generated as a major complication of hematological disorders (sickle cell disease (SCD), beta-thalassemia, chronic hemolytic anemia, myeloproliferative disorders or splenectomy), systemic disorders (sarcoidosis, pulmonary Langerhans cell histiocytosis, lymphangioleiomyomatosis, neurofibromatosis, or vasculitis), metabolic disorders (glycogen storage disease, Gaucher’s disease, or thyroid disorders), and other disorders such as chronic renal failure or tumoral obstruction [45,46].

Several mechanisms are thought to contribute to the pathophysiology of PH in hematological disorders. Sickle erythrocytes have shown high levels of ROS, which disrupt NO homeostasis [47]. Moreover, the impaired glutathione pathway and iron overload contribute to the increased ROS in SCD [47]. The overproduction of ROS promotes vasoconstriction and pulmonary vascular remodeling, resulting in the development of PH [47]. Novelli et al. [48] demonstrated that oxidative stress contributes to SCD-associated PH. They reported increased thrombospondin-1 levels, which contributed to ROS generation via binding to the CD47 receptor using a Berkeley model of SCD and patients with SCD-associated PH.

Oxidative stress also plays an important role in PH associated with thalassemia, which is exacerbated by hemolysis and the iron overload present in patients receiving transfusion therapy [49]. Additionally, alterations in the glutathione system in thalassemia make it difficult to remove reactive oxygen species in erythrocytes, contributing to their hemolysis. This increase in oxidative stress level, in addition to other pathogenic mechanisms, results in endothelial dysfunction and vascular damage [49].

It has also been reported that ROS levels of vascular ECs are increased in diabetes. The hyperglycaemic environment enhances EC permeability due to ROS-mediated upregulation of cell adhesion molecules exacerbating leukocyte adhesion and migration into the vascular wall, resulting in vascular damage [50].

In light of the observed involvement of ROS in the pathophysiology of PH, targeting these reactive species through the use of antioxidant therapy may present a viable and promising avenue for the treatment of PH.

**Table 2:** Studies linking oxidative stress with PH.

Type of PH	Organism	Oxidative Stress Biomarker	Molecular Changes	Effect on the Pathology	Treatment	Effect of the Treatment	Type of Study	Ref.
MCT-PAH	Rat	↑ROS ↓Catalase mRNA ↓GPX1 mRNA	↑Phosphorylation of PKM2 ↓PKM2 activity	↑PASMCs proliferation	NAC, apocynin, MnTBAP	↓ROS ↓Phosphorylation of PKM2 ↑PKM2 activity	In vivo/In vitro	[21]
PAH	Human	↑MAO-A expression		↑PVR	Clorgyline (MAO-A inhibitor)	↓MAO-A activity ↓ROS ↓PVR	In vivo/In vitro	[22]
PH-LHD	Rat	↑Peroxynitrite ↑O_2_-	↓PTEN expression	↑SMC proliferation ↑Vascular remodeling	HO-3867 (synthetic analog of curcumin)	↓Peroxynitrite ↓O_2_^−^ ↑PTEN expression ↓Vascular remodeling	In vivo/In vitro	[28]
Precapillary PH (Group I, III, IV and V)	Human	↑iPF_2α_-III	PGH_2_ stimulation	Pulmonary vessels constriction			In vivo/In vitro	[23]
HPH	Rat	↑PCOOH ↑XO activity		↑RVH ↑Pulmonary vascular thickening	NAC or Allopurinol	↓PCOOH ↓RVH ↓Pulmonary vascular thickening	In vivo/In vitro	[34]
HPH	Rat	↑NOX4 ↑VPO1 ↑HOCl	↑Expression of cell cycle regulators, apoptosis-related proteins, migration promoters, and NF-κB	Vascular remodeling ↑PASMCs proliferation, apoptosis resistance, and migration	BAY 11-7082 (an inhibitor of NF-κB)	↓Vascular remodeling ↓PASMCs proliferation, apoptosis resistance, and migration	In vivo/In vitro	[35]
CTEPH	Cell	↑ROS in CTEPH-EC ↑AOPPs ↑PCO ↓GPX4 and GPX1		Endothelial dysfunction			In vitro	[42]
CTEPH	Human	↑MDA ↓TAC activity ↓CAT activity		Adverse clinical outcomes			In vivo/In vitro	[37]
SCD-PH	Mouse/Human	↑TSP1 and CD47 expression ↑ROS		Endothelial dysfunction Promotion of PH in SCD	CD47 blockade	↓ROS ↓RV pressure ↓Mean pulmonary artery pressure	In vivo/In vitro	[51]

Abbreviations: PAH, pulmonary arterial hypertension; ROS, reactive oxygen species; GPX1, glutathione peroxidase 1; PKM2, pyruvate kinase M2; PASMC, pulmonary artery smooth muscle cells; NAC, N-acetylcysteine; MnTBAP, Mn(III) tetrakis (4-benzoic acid) porphyrin; MAO-A, monoamine oxidase A; PVR, pulmonary vascular remodeling; PH-LHD, pulmonary hypertension due to left heart disease; PTEN, phosphatase-and-tensin homolog on chromosome 10; SMC, smooth muscle cell; iPF2α-III, isoprostaglandin F2α type III; PGH_2_, thromboxane A2/prostaglandin H2 receptor; HPH, hypoxic pulmonary hypertension; PCOOH, phosphatidylcholine hydroperoxide; XO, xanthine oxidase; RVH, right ventricular hypertrophy; NOX4, NADPH oxidase subunit 4; VPO1, vascular peroxidase 1; HOCl, hypochlorous acid; NF-κB, nuclear factor kappa B; CTEPH, chronic thromboembolic pulmonary hypertension; AOPPs, advanced oxidation protein products; PCO, total protein carbonyl content; GPX4, glutathione peroxidase 4; MDA, malondialdehyde; TAC, total antioxidant capacity; CAT, catalase; SCD-PH, pulmonary hypertension (PH) associated with sickle cell disease (SCD); TSP1, thrombospondin-1. Upward arrows (↑) indicate an increase, and downward arrows (↓) indicate a decrease. All parameters have been measured in plasma, serum, pulmonary artery cells or lung tissues. Echocardiographic assessments and hemodynamic parameters, such as PVR, mPAP, or RVH, were measured in the heart.

## 4. Antioxidant Treatment

Oxidative stress can be mitigated by endogenous defense systems or treatment with exogenous antioxidant therapy. Antioxidants are substances that scavenge ROS, thus decreasing oxidative damage and maintaining cellular redox homeostasis [13]. The endogenous antioxidant defense system includes non-specific, nonenzymatic antioxidants, such as α-tocopherol (vitamin E), vitamin C, glutathione, uric acid, and bilirubin; and specific, enzymatic antioxidants, such as SODs, CAT, and GPx [13,51]. Exogenous antioxidant defense system includes carotenoids, flavonoids, and vitamins [13]. However, although antioxidant defense systems are widely distributed in the body, they are unequally distributed within cells, being located mainly in the cytoplasm and the mitochondria [13,51].

Redox therapeutic strategies follow the approach of targeting ROS to regulate oxidative stress levels for therapeutic benefit. This strategy has been applied in different diseases caused by oxidative stress, such as neurodegenerative and cardiovascular diseases (CVD) [12].

### 4.1. Global Antioxidants (Non-Targeted Antioxidant Treatments)

Due to the evident relationship between oxidative stress and the pathophysiology of PH, numerous studies have focused on testing the efficacy of global antioxidants in therapeutic strategies [52].

#### 4.1.1. Vitamins

Vitamin E is a potent antioxidant, scavenging peroxyl radicals, so preventing lipid peroxidation [53]. Peroxyl radicals are reduced to tocopheroxyl radicals, which are then reduced by vitamin C to regenerate vitamin E [52,54]. Vitamin C is a hydro-soluble vitamin that scavenges free radicals, it cannot be synthesized by the human body so must be taken as a supplement [52,54]. Vitamin D a fat-soluble vitamin available from dietary sources [55] and mainly synthesized endogenously in skin exposed to solar UVB radiation [55]. This produces an inactive precursor that is converted in the liver to 25-hydroxyvitamin D3 (25(OH)D3) (calcidiol) [55], then processed in the kidney to generate 1α, 25-dihydroxyvitamin D3 (1,25(OH)2D3), (calcitriol), the active metabolite of vitamin D [55].

There is extensive evidence of the relationship between the deficiency of some vitamins and PH development [56]. For instance, deficiency of vitamin C and iron, producing uncontrolled hypoxia-inducible factor activity and pulmonary vasoconstriction, has been related to reversible pulmonary hypertension [57]. Additionally, vitamin D deficiency was associated with a higher risk of mortality in PAH patients [58].

Several studies have reported that these vitamins have beneficial effects in many other diseases that involve oxidative stress, including CVD and cancer [52]. Callejo et al. [59] showed that restoration of optimal vitamin D levels improved endothelial dysfunction in animals with PAH. Vitamins D and E have also been shown to restore redox status by attenuating oxidative stress in PASMCs exposed to high glucose, a condition correlated with PH pathology. However, vitamin D supplementation did not ameliorate PVR or RVH [60].

#### 4.1.2. Melatonin

Melatonin, a neurohormone produced by the pineal gland, protects against ROS by direct scavenging of ROS, stimulation of antioxidant enzymes, and negative modulation of pro-oxidant agents [61,62]. Excessive ROS generation, the main cause of decreased vasodilator capacity and excessive proliferation of PASMCs, is associated with vascular dysfunction in PPHN. Studies have shown that in hypoxic models of PPHN, melatonin administration is effective in alleviating symptoms. Neonates are unable to produce melatonin, favoring ROS production in hypoxic conditions [62]. However, PPHN lambs treated with melatonin showed improved pulmonary vasodilation and decreased pulmonary arterial pressure, enhanced SOD and CAT expression, and decreased generation of superoxide anions and NADPH oxidases in RV [61,62]. In a rat model of hypoxic PH melatonin was shown to attenuate pulmonary pressure and vascular remodeling, decreasing XO activity, MDA levels, NOX4 expression, and increasing CAT, GPx and SOD activities [63].

#### 4.1.3. NAC

NAC, an N-acetyl derivative of the endogenous amino acid l-cysteine, is widely used as a mucolytic agent and to treat paracetamol overdose [64,65]. It also has neuroprotective properties and is used in neurodegenerative and psychiatric diseases [65]. NAC also generates anti-inflammatory effects by modulating the release of cytokines [65]. It is a thiol compound, so it can react with radical and non-radical oxidants, acting as a direct antioxidant, neutralizing free radicals [64,65]. It can also act as an indirect antioxidant, by increasing plasma cysteine levels, producing a subsequent increase in plasma GSH. NAC can also regulate the redox state by breaking disulphide bonds and has the capacity to restore thiol pools [64].

NAC has been proposed as a therapy for several disorders, including idiopathic pulmonary fibrosis, bronchitis, and lung ischemia–reperfusion injury [66]. In PH, administration of NAC to cultured PASMCs cells [67] and in animal models has been demonstrated to reduce oxidative stress levels and cardiopulmonary alterations [21,34,68].

These findings suggest that NAC may be an efficacious antioxidant treatment for PH, especially due to its demonstrated good tolerability, safety, and clinical efficacy [66]. A clinical trial evaluating the use of NAC in post-reperfusion pulmonary injury in patients with CTEPH is currently underway [69].

#### 4.1.4. Polyphenols

Polyphenols are a large group of plant compounds containing bioactive molecules present in the human diet, specifically in vegetables, fruits, and beverages [55,70]. Dietary polyphenols are classified as flavonoids, which comprises the major group of polyphenolic compounds, and non-flavonoid polyphenols [55,71]. Stilbenes, a non-flavonoid polyphenol, contain resveratrol that shows a variety of biological activities, including antioxidant, anti-inflammatory, and anti-tumor properties, and has been shown to improve PH [72]. Several studies have reported that resveratrol in MCT-induced PAH rats suppresses PASMC proliferation and pulmonary vessel muscularization [73,74]. The effects of resveratrol have been also studied in a CTEPH model, where it showed a reduction in vascular injury and pulmonary arterial pressure, not only by promoting SOD expression and scavenging ROS, but also inhibiting inflammation and platelet activation [72].

Additionally, a great number of studies have confirmed that flavonoids could prevent CVD and be a potential therapy as a result of their anti-inflammatory, anti-oxidant, and anti-proliferation activities [75]. Puerarin, one of the isoflavones, has demonstrated several pharmacological activities reducing hypoxia-induced pulmonary vascular remodeling and PASMC proliferation, so preventing the development of PH [76]. Puerarin also decreases ROS levels in human pulmonary artery ECs under a hypoxic conditions, protecting pulmonary arteries and could be a potential treatment for PH [77].

### 4.2. Mitochondria-Targeted Antioxidants

In the last few years, due to increasing evidence of the role of mtROS in several diseases, numerous mitochondria-targeted antioxidants have been studied for therapeutic intervention [13,78]. The accumulation of mtROS produces oxidative damage in intracellular lipids, DNA, and proteins, leading to the development of pathological conditions [79]. Therefore, current therapeutic strategies aim to develop drugs that restore mitochondrial function and modulate redox homeostasis [79]. Targeted delivery would allow antioxidants to reach elevated concentrations in mitochondria, whereas non-targeted antioxidants may be metabolized or inadequately absorbed before reaching mitochondria [79,80]. Mitochondria-targeted antioxidants are generally classified as lipophilic cation-based antioxidants, such as MitoQ, MitoVitE, MitoPBN, MitoPeroxidase, SkQ1, and SkQR1, or amino acid- and peptide-based antioxidants, such as SS tetrapeptides [80]. The advantage of these antioxidants for targeting mitochondrial ROS is their ability to cross the phospholipid bilayer and remove ROS from within the mitochondria [80].

A growing number of studies are using this targeted therapy in diseases involving mitochondrial oxidative damage such as neurological diseases, cardiovascular diseases, and cancer development [79].

#### 4.2.1. Lipophilic Cations

**Figure 2:**
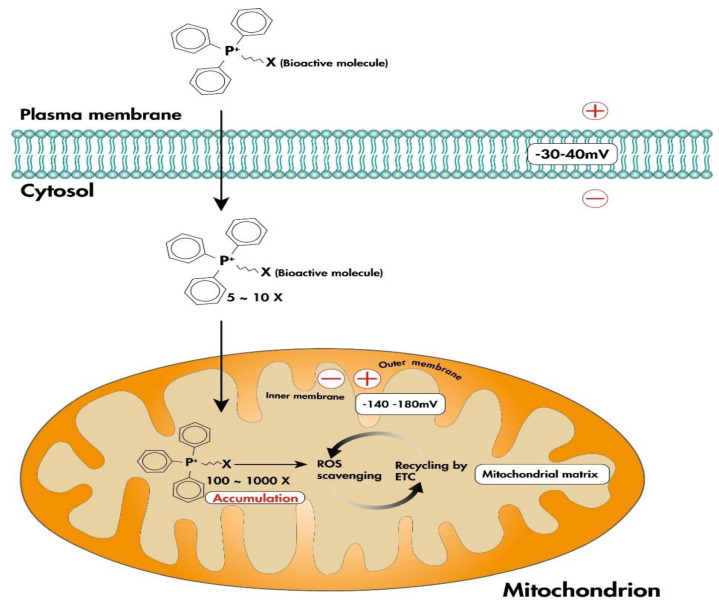
TPP-based mitochondria-targeted antioxidant. Linking the lipophilic cation TPP^+^ to the bioactive molecule allows the specific accumulation of the bioactive molecule within the mitochondria, acting as an antioxidant through scavenging mitochondrial ROS. The positive charge of TPP ^+^ allows it to rapidly pass through phospholipid bilayers and accumulate within the negatively charged mitochondrial matrix driven by the electrochemical gradient. Abbreviations: TPP^+^, triphenylphosphonium; ROS, reactive oxygen species; ETC, electron transport chain.

The main challenges in developing drugs that target the mitochondria are specificity of targeting and accumulation of the bioactive molecule in mitochondria [81]. One solution is to attach a lipophilic cation, such as triphenylphosphonium (TPP^+^), to the bioactive molecule in order to target it to the mitochondria (Figure 2) [81]. Lipophilic cations rapidly pass through the phospholipid bilayers and accumulate within the negatively charged mitochondrial matrix driven by the electrochemical gradient [13,81,82]. MitoQ and SkQ1 are the two most studied mitochondria-targeted antioxidants based on lipophilic cations [81].

##### MitoQ

MitoQ is an effective antioxidant against lipid peroxidation [82,83]. It consists of a derivative of ubiquinone conjugated to TPP^+^ that allows MitoQ to accumulate within the inner mitochondrial membrane [13,82], where it is reduced to the active antioxidant form, ubiquinol, by complex II of the respiratory chain. This active antioxidant is then oxidized to the inactive form, ubiquinone, which is continually recycled by complex II to its active form [82,83].

Preclinical studies have shown that 4 weeks of oral supplementation with MitoQ improves vascular endothelial function in old mice, mediated by mtROS reduction [84]. Cuevas et al. [85] translated these preclinical findings to humans, where they found an improvement in endothelial function and reduced aortic stiffness in participants exhibiting age-related aortic stiffening.

Additionally, the protective effects of MitoQ have also been reported in PH. Suresh et al. showed that treatment with MitoQ decreased ROS levels, ROS-induced Ca^2+^ influx and mitochondrial fragmentation, and reduced abnormal migration and proliferation of lung microvascular endothelial cells isolated from hypoxic rats [86,87]. Pak et al. [82] demonstrated that MitoQ inhibited mtROS production in PASMCs exposed to acute hypoxia, attenuating HPV, but did not inhibit the development of PVR and chronic hypoxia-induced PH.

##### SkQ1

SkQ1 is structurally similar to MitoQ and consists of a TPP^+^ unit conjugated to plastoquinone, a quinone present in chloroplasts [81]. This compound has been studied for its potential in treating various diseases, including age-related diseases, cancer, and cardiovascular disease.

Manskikh et al. [88] showed that the antioxidant SkQ1 prevented cardiac hypertrophy and fibrosis by lowering mtROS production and showed cardioprotective effects in aging mice. Similarly, SkQ1 administration ameliorated cardiomyocyte hypertrophy promoted by high fructose or H_2_O_2_, restoring mitochondrial metabolism [89]. Moreover, in endothelial cells, SkQ1 attenuates ICAM1, VCAM, and E-selectin expression, preventing leukocyte adhesion to the endothelial monolayer and the development of atherosclerosis [90].

#### 4.2.2. Peptide-Based Antioxidants

Szeto-Schiller (SS) peptides and the mitochondria-penetrating peptide (MPP) are peptide-based antioxidant delivery systems [79,91]. SS-peptides contain a single aromatic–cationic sequence motif, alternating between basic and aromatic residues, favoring efficient cell uptake, independent of membrane potential [79,91]. Aromatic residues provide necessary hydrophobicity and basic amino acids provide positive charge at physiological pH [92]. Similarly, MPPs consist of a combination of four to eight amino acids that alternate between positive charge and hydrophobic properties [91].

SS-31, also known as elamipretide, is a tetrapeptide with three positive charges that can easily penetrate cells and accumulate in the inner mitochondrial membrane [93,94]. It contains dimethyltyrosine residues that can react with oxidative radicals in mitochondria, providing strong antioxidant properties [93]. SS-31 has demonstrated great potential in the treatment of PH. In a transverse aortic constriction-induced PAH mouse model, SS-31 reduced right ventricular systolic blood pressure (RVSBP), preserved the architecture of the lung parenchyma and the RV and significantly increased the levels of antioxidants, improving PAH pathology [95].

XJB-5-131 is another MPP synthetic radical and electron scavenger. TEMPO, its antioxidant component, can mediate two-electron transfer processes, maintaining redox homeostasis. XJB-5-131 has been shown to reverse or attenuate disease progression. Polyzos et al. [96] showed that XJB-5-131 effectively decreases mtROS in the brain, preventing neuronal death.

While in vitro and preclinical in vivo studies have demonstrated favorable outcomes, clinical trials have largely failed to demonstrate any beneficial effects. Specifically, clinical trials utilizing non-targeted antioxidants have not shown beneficial effects to date. As a result, employing mitochondrial targeted antioxidants in treatments may prove to be a promising approach (Table 3).

## 5. Conclusions

In vitro and preclinical studies support a role for oxidative stress in the development of PH and have demonstrated the benefit of antioxidant targeting as a therapy. However, clinical studies continue to produce contradictory results. Here, we have discussed the role of oxidative stress in the development and progression of the different types of PH, which causes the proliferation of PASMC and EC, endothelial dysfunction, pulmonary vascular remodeling, and right ventricular hypertrophy. A number of global and targeted antioxidants have been used in PH models. However, the beneficial effects seen in preclinical studies are not reproduced in clinical application. Therefore, the continued exploration of antioxidant therapies is warranted to better understand the role of ROS in PH and to develop successful therapeutic interventions.

## Figures and Tables

**Table 1:** Updated classification of pulmonary hypertension.

**1.** **Pulmonary arterial hypertension**
1.1. Idiopathic (iHAP) 1.1.1. Non-responders at vasoreactivity testing 1.1.2. Acute responders at vasoreactivity testing 1.2. Heritable (hPAH) 1.3. Drug and toxin induced 1.4. Associated with 1.4.1. Connective tissue disease 1.4.2. HIV infection 1.4.3. Portal hypertension 1.4.4. Congenital heart diseases 1.4.5. Schistosomiasis 1.5. PAH with features of venous/capillary (PVOD/PCH) involvement 1.6. Persistent PH of the newborn
**2.** **Pulmonary hypertension caused by left heart disease**
2.1. Heart failure 2.1.1. with preserved ejection fraction 2.1.2. with reduced or mildly reduced ejection fraction 2.2. Valvular heart disease 2.3. Congenital/acquired cardiovascular conditions leading to post-capillary PH
**3.** **Pulmonary hypertension caused by lung diseases and/or hypoxia**
3.1. Obstructive lung disease or emphysema 3.2. Restrictive lung disease 3.3. Lung disease with mixed restrictive/obstructive pattern 3.4. Hypoventilation syndromes 3.5. Hypoxia without lung disease 3.6. Developmental lung disorders
**4.** **Pulmonary hypertension associated with pulmonary artery obstructions**
4.1. Chronic thrombo-embolic PH 4.2. Other pulmonary artery obstructions
**5.** **Pulmonary hypertension with unclear and/or multifactorial mechanisms**
5.1. Hematological disorders 5.2. Systemic disorders 5.3. Metabolic disorders 5.4. Chronic renal failure with or without haemodialysis 5.5. Pulmonary tumor thrombotic microangiopathy 5.6. Fibrosing mediastinitis

Abbreviations: HIV, human immunodeficiency virus; PAH, pulmonary arterial hypertension; PVOD, Pulmonary veno-occlusive disease; PCH, Pulmonary capillary hemangiomatosis; PH, pulmonary hypertension. Adapted from [4].

**Table 3:** Summary of recent clinical trials in PH, CVD, and other diseases that target oxidative stress.

Antioxidant Therapy	Condition	N	Study Design	Findings	Status	ClinicalTrials.gov Identifier
**Clinical trials in PH**						
NAC	CTEPH	34	Randomized clinical trial	No finding yet (still recruiting)	Recruiting	NCT04081012
CoQ10	PAH	18	Non-randomized clinical trial	Improved hemoglobin and red cell maturation	Completed	NCT01148836
BQ-123 with or without MitoQ or oral BH4	PAH	420	Non-randomized clinical trial	No finding yet (still recruiting)	Recruiting	NCT02966665
**Clinical trials in CVD**						
Vitamin D	CVD	80	Non-randomized clinical trial	VitD did not improve endothelial function, arterial stiffness, or inflammation	Completed	NCT01049048
Vitamin E and C	CVD	14,641	Randomized trial	No significant effect on cardiovascular events	Completed	NCT00270647
Melatonin	Smoke-induced Vascular Injury	68	Randomized clinical trial	Improved smoke-induced vascular injury	Completed	NCT02591238
NAC	Hypertrophic Cardiomyopathy	42	Randomized clinical trial	Small effect on cardiac hypertrophy or fibrosis	Completed	NCT01537926
Resveratrol	Peripheral Arterial Disease	66	Randomized clinical trial	No improvement in 6 MWT	Completed	NCT02246660
MitoQ	Peripheral Arterial Disease	13	Randomized clinical trial	No finding yet (still recruiting)	Recruiting	NCT03506633
**Clinical trials in other diseases**						
MitoQ	Chronic Obstructive Pulmonary Disease	24	Randomized clinical trial	No finding yet	Not yet recruiting	NCT05605548
SkQ1	Keratoconjunctivitis Sicca	91	Randomized clinical trial	Improved dry eye symptoms	Completed	NCT02121301

Abbreviations: PH, pulmonary hypertension; NAC, N-acetylcysteine; CTEPH, chronic thromboembolic pulmonary hypertension; CoQ10, coenzyme Q10; PAH, pulmonary arterial hypertension; CVC, cardiovascular disease; VitD, vitamin D; 6 MWT, 6 min walk test.
